# Supplementary material for: Annexin-V stabilizes membrane defects by inducing lipid phase transition
Source: Nat Commun. 2020 Jan 13;11:230. doi: 10.1038/s41467-019-14045-w (PMC6957514; doi:10.1038/s41467-019-14045-w)
Supplement: Supplementary file 1 — Supplementary Information [file 41467_2019_14045_MOESM1_ESM.pdf]

SUPPLEMENTARY INFORMATION

Annexin-V Stabilizes Membrane Defects by Inducing Lipid Phase Transition

Authors: Lin et al.

This PDF includes:

Supplementary Note 1

Supplementary Figures 1 to 3

Other Supplementary Information for this manuscript includes

Supplementary Movies 1 to 7

Supplementary Note 1

A5 2D-lattices confine high Ca^{2+}-ion concentrations to the membrane surface.

The tripartite A5 / Ca^{2+} / negatively-charged-lipid system sandwiches a layer of Ca^{2+}-ions between protein and lipid, functioning as a molecular glue for A5-membrane-binding. Thus, we engaged in the analysis of Ca^{2+}-binding sites among known A5 3D-structures (summarized in the Table below). Considering that 3 A5-trimers compose a unit cell in the A5-lattice (a=b=17.7nm; γ=60°), the unit area can be estimated as ~270 nm^2 housing 9 A5-monomers. Thus, under the assumption that Ca^{2+}-ions are sandwiched in a layer of ~1 nm thickness, the local Ca^{2+}-concentration near the membrane can be estimated between 110 mM (based on PDB 1BC0 with 2 Ca^{2+}-ions) and 606 mM (based on PDB 2H0K with 11 Ca^{2+}-ions), respectively. Our HS-AFM and MDS observations indicate that such tremendous Ca^{2+}-concentrations near the membrane alter the membrane properties towards more ordered phases.

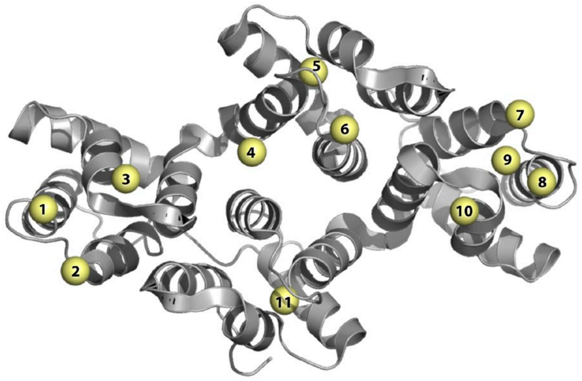

		Ca^{2+}-binding site										
		**1**	**2**	**3**	**4**	**5**	**6**	**7**	**8**	**9**	**10**	**11**
**PDB code**	**1ALA**	X				X						X
	**1AXR**	X	X	X			X					X
	**2RAN**		X	X		X			X		X	X
	**1ANW**	X										X
	**1ANX**	X		X				X	X			
	**1HXD**	X	X			X						X
	**1A8A**	X	X	X	X	X	X	X	X		X	X
	**1BC0**							X	X			
	**1N41**	X				X		X	X			X
	**2IE6**	X	X	X		X		X	X			
	**2H0K**	X	X	X	X	X	X	X	X	X	X	X

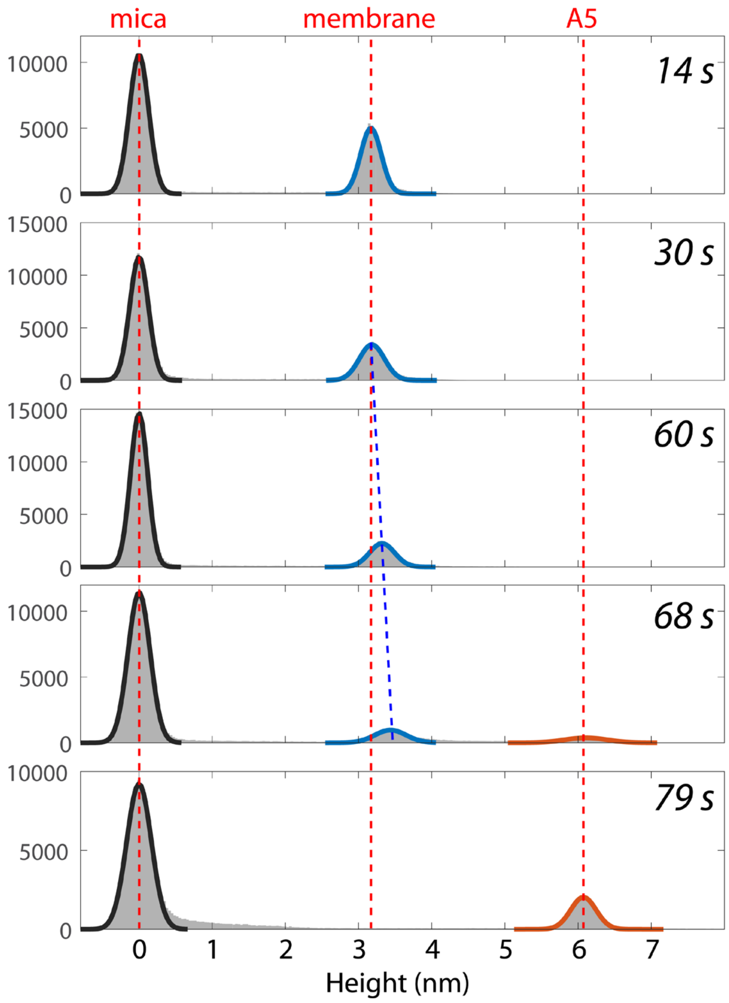

Supplementary Figure 1. Time-lapse height histograms of HS-AFM images shown in Figure 3c. For each HS-AFM frame, different number of Gaussian fits (two or three) was used to analyze the mean height of mica (black), membrane (blue), and A5 (orange), respectively. The blue dashed line indicates the increase of membrane thickness following A5 membrane adsorption and during the self-assembly process.

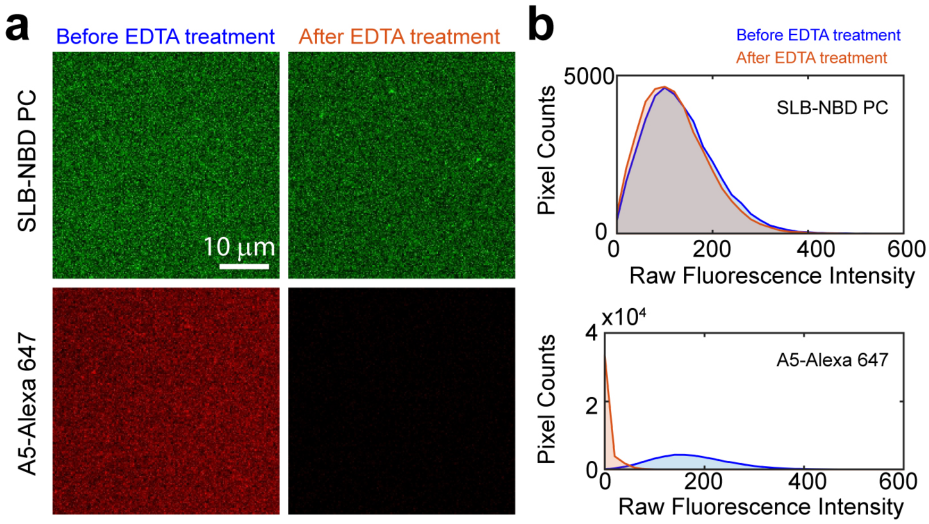

Supplementary Figure 2. Removal of A5 molecules from the SLB using EDTA during FRAP experiments. (**a**) Representative fluorescence images of SLB membrane and A5 molecules recorded before and after EDTA treatment. Two separate spectral channels with different excitation lasers were used to monitor NBD-PC and A5-Alexa 647, respectively. (**b**) The histograms of raw fluorescence intensity calculated from images in (a). EDTA-addition can successfully remove A5 molecules form the SLB with an ignorable impact on fluorescent NBD-PC in SLB.

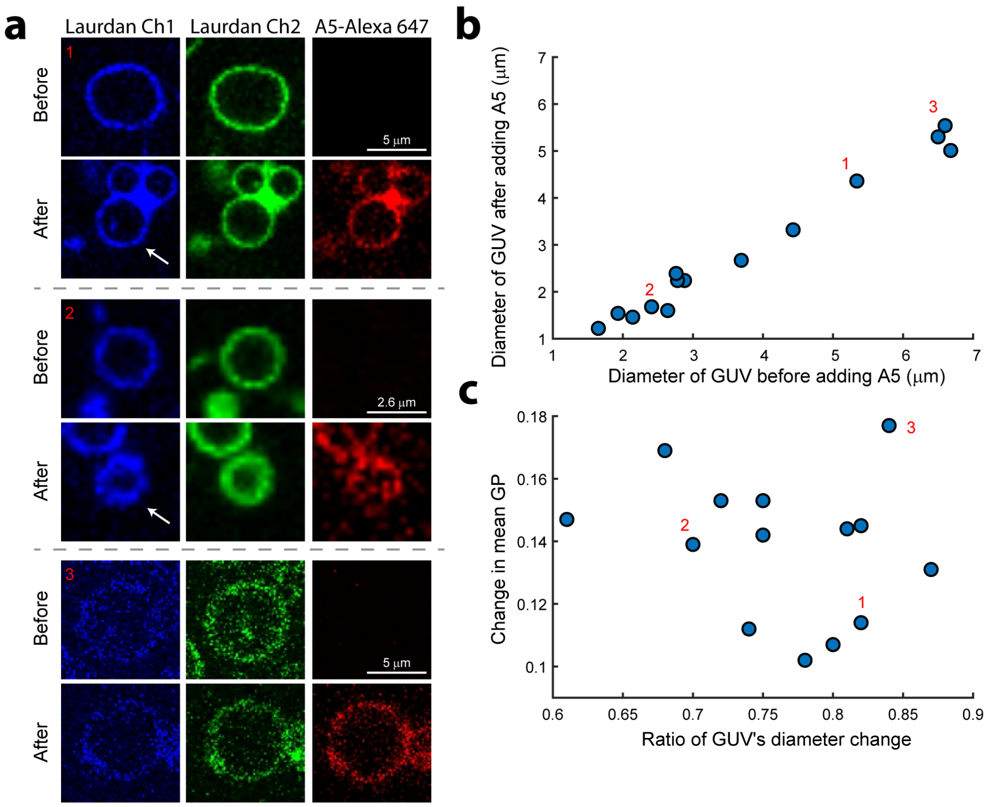

Supplementary Figure 3. Analysis of GUV diameter before and after A5 binding. (**a**) Representative CLSM fluorescence images showing the impact of A5 2D-lattice self-assembly on GUVs. (**b**) The correlation of GUV diameter before and after A5 addition. (**c**) The correlation between diameter change and GP value change induced by the A5 2D-lattice self-assembly on GUVs. We note that most free-standing GUVs ruptured or moved away from the monitored region during the additions of A5, due to flow. Thus, only the GUVs that were clearly identified before and after A5-addition are reported here. When GUVs aggregated, we used partial, non-overlapping regions of the GUVs to calculate the mean GP value.
