# Supplementary material for: Annexin-V stabilizes membrane defects by inducing lipid phase transition
Source: Nat Commun. 2020 Jan 13;11:230. doi: 10.1038/s41467-019-14045-w (PMC6957514; doi:10.1038/s41467-019-14045-w)
Supplement: Supplementary file 9 — Description of Additional Supplementary Files [file 41467_2019_14045_MOESM9_ESM.docx]

**Description of Additional Supplementary Files**

**File Name:** Supplementary Movie 1.

**Description:** Boarder dynamics of a finite A5 2D-lattice on a membrane patch (Figure 2b) recorded by HS-AFM at 400nm x 400nm image size and 0.3s per frame.

**File Name:** Supplementary Movie 2.

**Description:** The formation of a supported lipid bilayer membrane on the mica substrate through vesicle delivery (Figure 3a) recorded by HS-AFM at 700nm x 700nm image size and 0.94s per frame.

**File Name:** Supplementary Movie 3.

**Description:** The movement and fusion of two small membrane patches (Figure 3b) recorded by HS-AFM at 375nm x 375nm image size and 1s per frame.

**File Name:** Supplementary Movie 4.

**Description:** The impact of the A5 2D-lattice growth on the finite membrane patch (Figure 3c) recorded by HS-AFM at 445nm x 510nm image size and 1s per frame.

**File Name:** Supplementary Movie 5.

**Description:** The formation of membrane around A5-protected membrane patches through vesicle delivery (Figure 7a) recorded by HS-AFM at 750nm x 750nm image size and 1s per frame.

**File Name:** Supplementary Movie 6.

**Description:** A5 dynamics around a membrane defects following the addition of supplemental A5 to the HS-AFM fluid cell (Figure 7c, following Supplementary Movie 5) recorded by HS-AFM at 400nm x 400nm image size and 0.2s per frame.

**File Name:** Supplementary Movie 7.

**Description:** A5 2D-lattice dynamics around a membrane defect (Figure 7d, following Supplementary Movie 6) recorded by HS-AFM at 150nm x 150nm image size and 0.2s per frame.
